# Photoluminescence and Photocatalytic Properties of MWNTs Decorated with Fe-Doped ZnO Nanoparticles

**DOI:** 10.3390/ma16072858

**Published:** 2023-04-03

**Authors:** Adriana Popa, Maria Stefan, Sergiu Macavei, Laura Elena Muresan, Cristian Leostean, Cornelia Veronica Floare-Avram, Dana Toloman

**Affiliations:** 1 National Institute for Research and Development of Isotopic and Molecular Technologies, 67-103 Donat, 400293 Cluj-Napoca, Romania; adriana.popa@itim-cj.ro (A.P.); cristian.leostean@itim-cj.ro (C.L.);; 2 Raluca Ripan Institute for Research in Chemistry, Babes-Bolyai University, 30 Fântânele, 400294 Cluj-Napoca, Romania; laura.muresan@ubbcluj.ro

**Keywords:** nanocomposites, photoluminescence, photocatalysis

## Abstract

The present work reports the photoluminescence (PL) and photocatalytic properties of multi-walled carbon nanotubes (MWCNTs) decorated with Fe-doped ZnO nanoparticles. MWCNT:ZnO-Fe nanocomposite samples with weight ratios of 1:3, 1:5 and 1:10 were prepared using a facile synthesis method. The obtained crystalline phases were evidenced by X-ray diffraction (XRD). X-ray Photoelectron spectroscopy (XPS) revealed the presence of both 2+ and 3+ valence states of Fe ions in a ratio of approximately 0.5. The electron paramagnetic resonance EPR spectroscopy sustained the presence of Fe^3+^ ions in the ZnO lattice and evidenced oxygen vacancies. Transmission electron microscopy (TEM) images showed the attachment and distribution of Fe-doped ZnO nanoparticles along the nanotubes with a star-like shape. All of the samples exhibited absorption in the UV region, and the absorption edge was shifted toward a higher wavelength after the addition of MWCNT component. The photoluminescence emission spectra showed peaks in the UV and visible region. Visible emissions are a result of the presence of defects or impurity states in the material. All of the samples showed photocatalytic activity against the Rhodamine B (RhB) synthetic solution under UV irradiation. The best performance was obtained using the MWCNT:ZnO-Fe(1:5) nanocomposite samples, which exhibited a 96% degradation efficiency. The mechanism of photocatalytic activity was explained based on the reactive oxygen species generated by the nanocomposites under UV irradiation in correlation with the structural and optical information obtained in this study.

## 1. Introduction

Water pollution caused by chemicals or microorganisms is a major threat to human health and the aquatic environment. Industrialization and agricultural activities are the main causes of water contamination, releasing various solvents or organic and inorganic compounds into the water. Among the pollutants discharged into water, organic dyes used in activities performed by the textile, paper or cosmetic industries are a class of major environmental pollutants due to their high toxicity [1]. Annually, approximately 7 × 10^7^ tons of dyes, such as azo, sulfide, direct, reactive, basic and acid dyes, are fabricated [2]. For example, Rhodamine B is an azo dye widely used in the textile, plastic and leather industries, which, in high concentrations, can constitute a health risk due to its carcinogenic and neurotoxic effect [3]. Another synthetic azo dye is methyl orange (MO), which is generally used as a coloring agent in the textile and leather industries [4]. It is widely used in printing, paper manufacturing, pharmaceutical, food processing industries and in research laboratories [5,6]. MO is soluble in water, and it can cause vomiting and diarrhea. High concentrations of MO can cause death [7]. It is stable and has low biodegradability; hence, it is difficult to remove from aqueous solutions using common water purification or treatment methods [6]. Among the reported recalcitrant dyes, Remazol brilliant blue (RBB) was classified as toxic, carcinogenic and extremely harmful to aquatic and vegetative lives [8]. RBB dye is used in the textile industry to fabric nylon, wool and silk. There are many studies on the toxicity of dyes and their impact on the ecosystem [9]. The challenge remains to successfully degrade this toxic dye from the aqueous system with minimum energy and without harming the ecosystem. Over time, researchers have attempted to find effective solutions to surmount these problems. Conventional methods such as adsorption, coagulation, ion flotation [10], sedimentation [11] and ozone electrolysis [12] have led to satisfactory results, but present some shortcomings, such as [13] the large consumption of chemical reagents, generation of secondary waste products and high costs [14]. The advanced oxidation process (AOP) provides an efficient alternative to the efficient elimination of dyes from water through reactive oxygen species (ROS) generation [15]. Among the AOP methods, photocatalysis mediated by metal oxide semiconductors has been intensively studied in recent years to make the process more efficient and to avoid the appearance of by-products. This process involves exciting the semiconductor with light, resulting in electrons transfer from the valence band (VB) to the conduction band (CB), leaving a hole in the VB. If the photogenerated charges do not recombine, they can generate ROS by interacting with water molecules. ROS are reactive species able to degrade a wide variety of pollutant molecules.

In recent years, wide interest has been attributed to composite materials based on MWCNTs and oxide semiconductors as photocatalysts for water purification applications. MWCNTs, due to their large specific surface area, play a supporting role in photocatalyst. However, it has been shown that the role of nanotubes is not only to support the photocatalytic material, but can also lead to a better separation of photogenerated charges [16]. Due to its high electron storage capacity and good conductivity, MWCNTs can receive photogenerated electrons from the semiconductor material, leading to the efficient separation of e^−^/h^+^ pairs. Moreover, MWCNTs can also play the role of a photosensitizer by increasing the photogenerated charge density of a semiconductor by releasing its electrons into the semiconductor’s CB [17]. The synergistic effects between MWCNTs and several types of oxide semiconductors, such as TiO_2_ [18], WO_3_ [19], ZnO [15], TiO_2_-Fe_3_O_4_ [20], BiOI [21], MIL-101(Fe) [22] and ferrites [23], which achieve the efficient degradation of antibiotics or dyes, were reported. Another strategy to streamline photocatalytic activity is doping semiconductors with rare earth or transition metal ions [24,25,26,27,28]. Doping increases the ability of semiconductors to adsorb light due to the decrease in the band gap through the introduction of new energy levels inside the band gap. Moreover, altering the relative Fermi-level density of states will enhance the excitation of photogenerated electrons [29].

In this work, we aim to develop a highly efficient composite material in RhB pollutant degradation under UV light irradiations. A facile chemical method was developed to obtain nanoparticles with a controllable morphology. Additionally, using PAH to attach ZnO:Fe nanoparticles along the MWCNT represents a new strategy to obtain MWCNT-ZnO nanocomposites without other chemical modifications that could destroy the structure of the MWCNT. We studied the synergistic role of MWCNT addition and Fe doping on the ZnO photocatalytic properties. In addition, Fe ion doping generates defect states that are able to delay the photogenerated charge recombination, favoring the photocatalytic degradation process. Correlating the XPS, PL and EPR trapping technique results, a photodegradation mechanism was proposed.

## 2. Materials and Methods

### 2.1. Materials

For the preparation of MWCNT-ZnO:Fe0.5%, we used the following materials and reagents: multi-walled carbon nanotubes (MWCNTs) with a 99% purity were purchased commercially (Sigma-Aldrich, Merck, KGaA, Darmstadt, Germany), poly-allylamine hydrochloride (PAH) (Alfa–Aesar, Thermo Fisher, Kandel, Germany), sodium chloride (NaCl)-(Alpha Aesar, Bio Aqua Group, Targu Mures, Romania), zinc nitrate hexahydrate (Zn(NO_3_)_2_·6H_2_O) (Alpha Aesar, Bio Aqua Group, Targu Mures, Romania), iron nitrate nonahydrate (Fe(NO_3_)_3_·9H_2_O) (Alpha Aesar, Bio Aqua Group, Targu Mures, Romania), absolute ethanol (C_2_H_5_OH-EtOH) (Alpha Aesar, Bio Aqua Group, Targu Mures, Romania). All of the chemicals are of analytical grade and used without further purification. The aqueous solutions were prepared with Milli-Q water from the Direct-Q 3UV system (Millipore, Bedford, MA, USA). Dimethyl sulfoxide (DMSO; >99.9%) was purchased from VWR Chemicals, and 5,5-dimethyl-1-pyrroline N-oxide (DMPO; >97%), dimethylformamide (DMF) and Rhodamine B were purchased from Sigma-Aldrich, Merck, KGaA, Darmstadt, Germany.

### 2.2. Sample Preparation

#### 2.2.1. Synthesis of ZnO:Fe Nanoparticles

The 0.5 mol% Fe-doped ZnO NPs—ZF0.5% were synthesized using the chemical precipitation method. The experimental procedure was performed according to our previous paper [30], with modifications to the reagent’s concentration and temperature. Thus, stoichiometric amounts, consisting of (3−x) g of Zn(NO_3_)_2_·6H_2_O (98%) and x g Fe(NO_3_)_2_·9H_2_O (x represents 0.5mol% from Zn(NO_3_)_2_·6H_2_O), were dissolved in 100 mL of ultrapure water and mixed under vigorous stirring to form a homogeneous solution. Subsequently, a solution of 3 M NaOH was added dropwise at a constant stirring rate until a white precipitate of zinc hydroxide was obtained. After pH = 12 was reached, the mixture was continuously stirred for 4 h at room temperature. The obtained ZF0.5% was washed with ultrapure water and dried at 65 °C for 24 h. A doping of 0.5 mol% was chosen because it presented the best photocatalytic activity in the tests performed on the Zn_1−x_Fe_x_O (x = 0, 0.1, 0.3, 0.5, 0.7) nanoparticles (Figure S1).

#### 2.2.2. Decoration of MWCNT with ZnO:Fe

The decoration of MWCNTs with ZF0.5% nanoparticles was achieved through polymer wrapping using poly(allylamine hydrochloride) (PAH). The role of PAH is to provide functional groups (amine, OH) on the MWCNT surface, as well as stability. The MWCNTs (10 mg) were dispersed in a 0.5 wt% PAH salt solution (0.5 M NaCl, 500 mL), sonicated for 3 h, then stirred overnight at 80 °C. The separation of the MWCNT-PAH was performed by centrifugation, followed by re-dispersion in water. Before the decorating process, ZF0.5% nanoparticles were dispersed separately through sonication in ethanol for 30 min and then mixed together for another 4 h. Three different samples based on the MWCNTs and ZF0.5% were prepared, in which the molar ratio between the MWCNTs and ZF0.5% was 1:3, 1:5 and 1:10, correspondingly denoted as CZF1:3, CZF1:5 and CZF1:10.

### 2.3. Methods

X-ray diffraction was performed using a Rigaku-SmartLab automated Multipurpose X-ray Diffractometer with Cu-Kα radiation, operating at 45 kV, 200 mA and using a D/tex Ultra 250 detector monochromator with XRF reduction. The morphology of the composite nanoparticles was determined through scanning transmission electron microscopy (STEM). The STEM analysis was achieved using a Hitachi SU8230 microscope (Tokyo, Japan), provided with a cold field emission gun that accelerates the electron at 200 kV. The UV–Vis characterization was conducted using a JASCO V570 UV–Vis–NIR Spectrophotometer equipped with an absolute reflectivity measurement JASCO ARN-475 accessory (Easton, MD, USA). The obtained reflectance spectra were transformed in absorbance using the intern soft of the spectrophotometer. The luminescent characteristics of the samples were evaluated based on the emission spectra registered with a JASCO FP-6500 spectrofluorometer Wavell equipped with a PMT R928 photomultiplier (glass filter WG 320-ReichmannFeinoptik). X-Ray Photoelectrons Spectroscopy (XPS) was used to determine the Fe chemical state with a SPECS custom-built system using the Mg anode (1253.64 eV). The CASA software 2.3.21 was used for spectrum analysis. Electron paramagnetic resonance (EPR) spectroscopy measurements of powder samples were carried out using a Bruker E-500 ELEXSYS spectrometer (Karlsruhe, Germany.) at room temperature under identical conditions: The measurements were conducted in both X-band (9.52 GHz) and Q band (33.9 GHz), microwave power 2 mW, modulation frequency of 100 kHz. For the EPR spectra simulation, an Anisotropic SpinFit Bruker was used. To monitor the reactive oxygen species (ROS) generation, the EPR Bruker E-500 ELEXSYS X-band (9.52 GHz) spectrometer coupled with the spin trapping probe technique was used. DMPO was utilized as a spin trapping reagent. The nanoparticles (10 mg) were dispersed in DMSO (1 mL) and homogenized in an ultrasound bath (30 min) before use. DMPO of 0.2 mol/L concentration was added to the suspension. The samples were prepared immediately before measurement and transferred into the quartz flat cell, optimized for liquid measurements. A high-performance liquid chromatography-Mass spectrometry (HPLC/MS) equipped with an electrospray ionization (ESI) positive ion mode was used to determine the reaction products. The HPLC-UV system was used to separate the intermediary compounds, which were identified by soft ionization mass spectrometry (ESI–MS). The mobile phase used was methanol (solvent A) and HPLC water containing 0.1% formic acid (solvent B) (60–90% methanol over 30 min).

### 2.4. Evaluation of Photocatalytic Activity

The photocatalytic activity was tested by evaluating the degradation of Rhodamine B (RhB) under UV light irradiation. A laboratory reactor consisting of two UV lamps (15 W) emitting at 365 nm and a quartz reaction vessel was used for this purpose. The catalyst (2 mg) was suspended in an aqueous solution of RhB (1.0 × 10^−5^ mol/l, 10 mL), and then the mixture was magnetically stirred in the dark to achieve the adsorption equilibrium. Each degradation experiment was continuously conducted for 150 min. The mixture (3.5 mL) was withdrawn for analysis every 60 min and separated from the suspensions through centrifugation. The analysis was performed using a UV–Vis spectrophotometer by recording the maximum absorbance of RhB at 554 nm. The photocatalytic activity was calculated using the equation:

Photocatalytic activity (%) = (1 − A_t_/A_0_) × 100 (1)

where A_t_ and A_0_ represent the RhB absorbance located at 554 nm at time t and at time t = 0, respectively.

## 3. Results and Discussions

**Figure 1:**
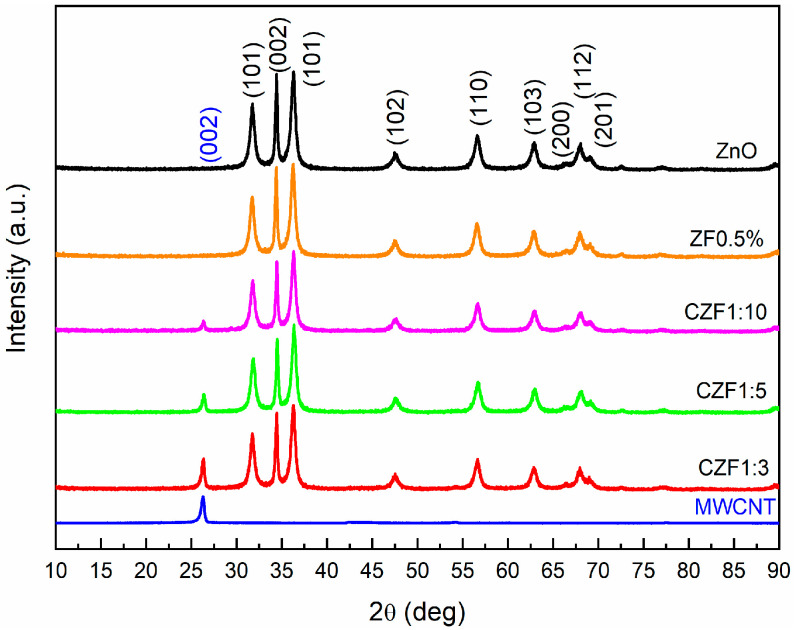
XRD diffraction patterns of the samples.

The structural characterization of the samples was achieved using XRD and EPR spectroscopy. The XRD diffraction patterns of the MWCNT, ZnO, ZF0.5% nanoparticles and CZF composite samples are illustrated in Figure 1. The diffraction spectrum of the MWCNT shows a (002) peak specific to standard graphite carbon (ICDD card 01-075-0444).

The diffraction patterns of ZF0.5% fit very well with those of ZnO, with a hexagonal structure (ICDD 01-080-0075). In the case of the nanocomposite samples, only the peak (002) specific to the MWCNT is observed, in addition to the patterns specific to ZnO. This peak decreases with the increase in the ZF0.5% content. The ratio between the peak intensity I_002_(MWCNT) and I_002_(ZnO) decreases from 0.36, in the case of the 1:3 sample, to 0.12, in the case of the 1:10 sample. Using Rietveld refinement, the lattice parameters, unit cell volume and the mean crystallites size were evaluated for the ZF0.5% and ZnO nanoparticles. The obtained parameters are presented in Table 1.

The lattice parameters of the ZF0.5% nanoparticles (a = 3.248 Å, and c = 5.206 Å) are close to those of the ZnO nanoparticles (a = 3.247 Å, and c = 5.202 Å), resulting in a slight increase in the unit cell volume, up to 47.56 Å^3^ from 47.50 Å^3^. Comparing the ionic radius of Fe^2+^ (0.76 Å), Fe^3+^ (0.64 Å) and Zn^2+^ (0.74 Å), it is probable that both Fe ions enter into the lattice of ZnO in a substitutional position. Through doping, a slight broadening of the peaks can be observed, leading to a smaller mean crystallites size of 11.5 nm for the ZF0.5% nanoparticles compared with 13.4 nm for the ZnO nanoparticles. Similar behavior was reported in the literature, and was attributed to the reduction in the ZnO nucleation rate caused by the dopant ions [31].

**Figure 2:**
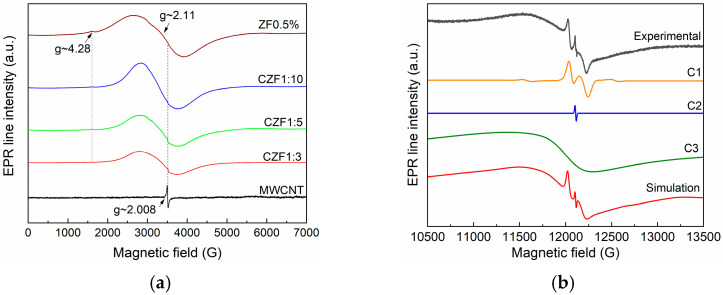
(**a**) X band spectra of MWCNT, ZF0.5% and CZF1:x (x = 3, 5, 10); (**b**) Q band spectra experimental and simulated spectrum of ZF0.5%. The simulated spectrum resulted from the sum of C1, C2 and C3 components.

EPR characterization was carried out to determine the dopant ions’ location and valence state. Figure 2a shows the room temperature EPR spectra measured in the X band of the MWCNT and ZF0.5%, which were compared with the spectra corresponding to the composite samples. The EPR spectrum of the MWCNT is composed of an intense and narrow line (ΔH = 17G) at g~2.008, usually attributed to carbon radicals. The formation of these radicals is a consequence of the MWCNT backbone breaking caused by ultrasonic treatment [32]. The ZF0.5% spectrum comprises a low intense line at g~4.28 and a broad line at g~2.11. Due to their large zero-field splitting and low spin-lattice relaxation time, the Fe^2+^ ions did not provide an EPR spectrum at room temperature [33]. Consequently, the obtained EPR line could be assigned to the Fe^3+^ ions. The line at g~4.2 is usually attributed to the presence of a Fe^3+^ ion (S = 5/2) located near the nanoparticle surface in a rhombically distorted octahedral position. Similar behavior was reported in other Fe-doped ZnO nanoparticles [33].

A closer analysis of the EPR signal from g~2.11 indicates that it is composed of several overlapping lines. This sample was also measured in the Q band (33.9 GHz) to better separate these contributions. The obtained spectrum is presented in Figure 2b. After performing the simulations, three contributions were identified: (i) isolated Fe^3+^ (S = 5/2, g~2.006, ΔH = 90G, D = 620 G–C1) situated in the nanoparticles core, substituting the Zn ions [34,35]; (ii) oxygen vacancies (g~2.004, ΔH = 6 G–C2) located mainly on the nanoparticles surface; (iii) an intense and large line (g~2.1, ΔH = 940G–C3), which is due to ferromagnetically coupled Fe^3+^ ions [36].

The EPR spectra of the composite material are composed by overlapping the MWCNT-specific spectrum and that of the Fe-doped ZnO. As the concentration of the Fe-doped ZnO increases (1–3 to 1–10), its contribution to the EPR spectrum becomes predominant, and the MWCNT-specific line decreases in intensity.

**Figure 3:**
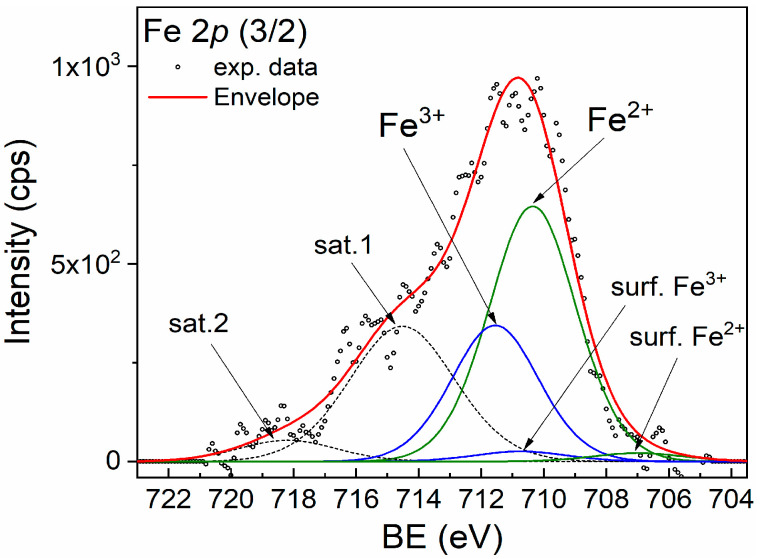
XPS spectrum of Fe *2p* (3/2) line corresponding to sample CZF1:3.

For better identification of the Fe chemical states, XPS spectroscopy was performed. The XPS spectra of the Fe *2p* (3/2) of the CZF1:3 sample are shown in Figure 3. The broad peak suggests that both Fe^2+^ and Fe^3+^ chemical states are present. The main peaks at 710.35 eV and 711.55 eV are attributed to the Fe^2+^ and Fe^3+^ chemical states, respectively. The corresponding shake-up satellites are positioned at 714.52 eV and 718.19 eV. In addition, small quantities of surface states with lower binding energy are present at 706.98 eV (Fe^2+^) and 710.74 eV (Fe^3+^) [33]. The Fe^3+^/Fe^2+^ ratio is 0.5.

**Figure 4:**
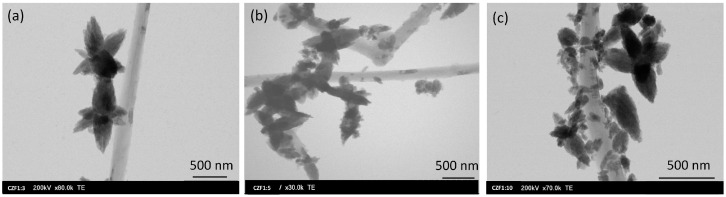
TEM images of CZF1:x ((**a**) x = 3, (**b**) x = 5, (**c**) x = 10) samples.

The morphology of the CZF1:x (x = 3, 5, 10) nanocomposites was evaluated using TEM, as presented in Figure 4. The representative images show the tubular shape of the MWCNTs decorated with ZF0.5% nanoparticles, which possess a star-like morphology, with a pronounced orientation depending on the nucleation and crystal growth. Figure S2 shows the size distribution of the ZF0.5% nanoparticles considering the length of the star petals. The mean size is approximately 210 nm. Considering the crystallite size determined by the XRD analysis (11.5 nm), the polycrystalline nature of the nanoparticles is evidenced. It is assumed that after the link between the PAH and the ZnO nanoparticle is formed, the attached nanoparticle becomes a crystallization center for the other nanoparticles from the solution. In our specific case, the experimental parameters favor this oriented growth. As expected, the grafting of the ZF0.5% nanoparticles along the MWCNT is dependent on the quantity of nanocomposites in the composite samples.

**Figure 5:**
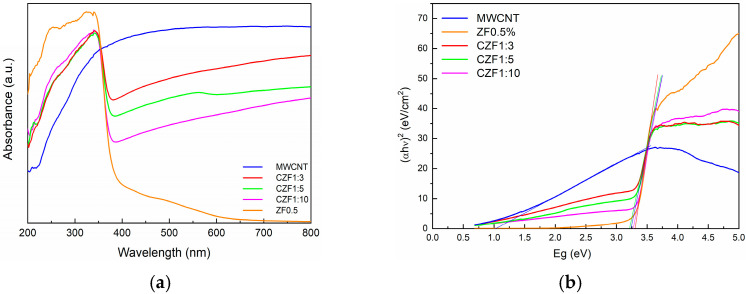
(**a**) UV-Vis absorption spectra of the sample. (**b**) Tauc’s plots.

Optical absorption spectroscopy provides information about the energy gap (E_g_) and bond structure based on the analysis of optically induced transitions. In the absorption process, through the absorption of a photon with a specified energy, an electron transition from a lower to a higher energy state occurs. Both reflectance and UV/Vis spectroscopy use UV-visible light to excite valence electrons to empty orbitals. The difference is that, in diffuse reflectance, we measure the relative change in the amount of light reflected by a surface, whereas in UV/Vis spectroscopy, we measure the relative change in the transmittance of light as it passes through a solution. UV–Vis diffuse reflectance spectroscopy (DRS) was used to determine the optical absorption properties of the samples. Figure 5a illustrates the UV-Vis absorption of the prepared samples. The MWCNT shows a broad absorption in all of the investigated ranges, with a maximum in the visible domain, while the ZF0.5% nanoparticles show strong absorption in the UV region, with a maximum absorbance at the 330 nm wavelength corresponding to the inter-band transition.

The low and broad absorption in the 400–600 nm wavelength range is due to the interfacial charge transfer and d–d transition between the multiplets of the 3d^5^ configuration of the high spin Fe^3+^ substituting Zn^2+^ under the influence of the tetrahedral ZnO crystal field. The observed transitions were assigned to the ^6^A_1g_ ground state to ^4^T_1_, ^4^T_2_, ^4^E and ^4^A_1_ excited states [37,38].

In the CZF1:x composite samples, the contribution of both components can be observed and is correlated with the ratio between them. The increase in the ZnO content leads to a decrease in the intensity of the visible light absorption. Based on the absorption spectra and using the Tauc’s equation [39], the band gap energy of the samples was evaluated. The Tauc’s plots are presented in Figure 5b. The band gap energy of the ZF0.5% nanoparticles is 3.30 eV. For the composite samples, due to the MWCNT contribution, the band gap energy decreases from 3.29 eV, in the case of the CZF1:10 sample, to 3.20 eV in the case of the CZF1:3 sample.

**Figure 6:**
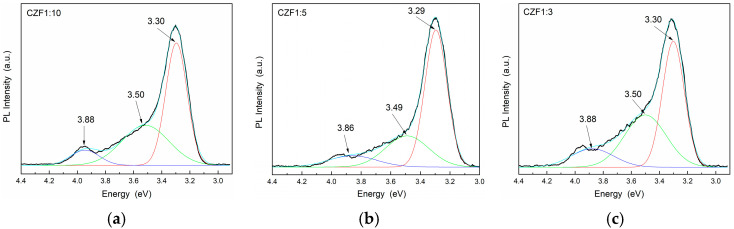
PL emission spectra of the samples (**a**) CFZ1:10, (**b**) CFZ1:5 and (**c**) CFZ1:3 under an excitation wavelength of 270 nm.

The photoluminescence properties provide information about the presence of defects or impurities and the efficiency of the charge carrier recombination. The emission spectra and the Gaussian deconvolution of the composite samples obtained under an excitation wavelength of 270 nm are shown in Figure 6. Table 2 summarizes the results related to the area of the peaks obtained after the spectra deconvolution.

In the range 300–400 nm (4.13–3.10 eV), there are three emission bands, known as a near band edge (NBE), due to the recombination of electrons from the CB with holes from the VB of ZnO [40]. For all three peaks, a quench of the emission can be observed by increasing the MWCNT content, probably due to a transfer of photoexcited charges from the ZnO to the MWCNT empty states [41,42].

**Figure 7:**
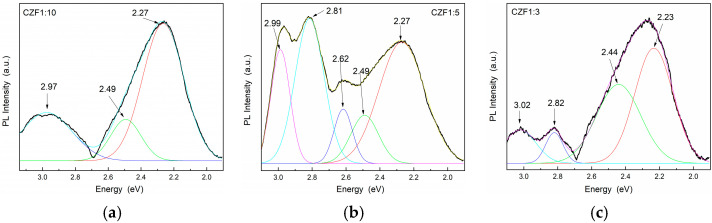
PL emission spectra of the samples (**a**) CFZ1:10, (**b**) CFZ1:15 and (**c**) CFZ1:3 under an excitation wavelength of 360 nm.

The lowest UV emissions were observed for CZF1:5, which means that this sample shows a longer delay in the recombination process. Moreover, a Stokes shift of about 0.35 eV can be observed between the maximum of the PL emission spectra and that of the absorption spectra, which can be a result of different effects, such as interface and point defects, which give rise to a red shift of the emission bands from the absorption edge [43]. To evidence the emission bands from the visible domain, the emissions spectra were obtained under an excitation wavelength of 360 nm. The obtained PL spectra of the composite samples and their Gaussian deconvolution are shown in Figure 7. Table 3 summarizes the results related to the area of the peaks obtained after the spectra deconvolution.

In the violet range, the composite samples exhibit one emission at 2.99 eV (415 nm). In the literature, there are two scenarios in the assignment of this band. Some authors attribute this band to: (i) Zn atoms in interstitial sites (Zn_i_), specific to an electronic transition between the Zn_i_ level and oxygen vacancies [42,44,45], and others to: (ii) unintentional H dopants, which can appear in ZnO samples prepared using chemical precipitation methods [46,47]. Indeed, in the synthesis process used in our samples, the PAH binding polymer was used to attach Fe-doped ZnO nanoparticles onto the surface of the MWCNT. The protonated functional groups from the PAH structure should contribute to these states. The blue emissions centered at 2.82 eV (439 nm), or 2.62 eV (473 nm), were attributed to the transition between the Zn_i_ level and V_Zn_ or to surface deep trap states [48]. The green emission bands centered at 2.5 eV (496 nm) and 2.21 eV (560 nm) were assigned to the transitions from Zn_i_ to the oxygen vacancies. The two green emissions can arise from different shallow energy levels in direct proximity to the band gap associated with the interface trap in the grain boundaries and dislocations [49]. From the analysis of the data obtained after the spectra deconvolution (Table 2) results, as in the case of the UV emissions, those in the visible range are quenched with the increase in the MWCNT content due to a delay In the recombination process induced by the charge transfer between defects levels of the ZnO nanoparticles and MWCNT.

**Figure 8:**
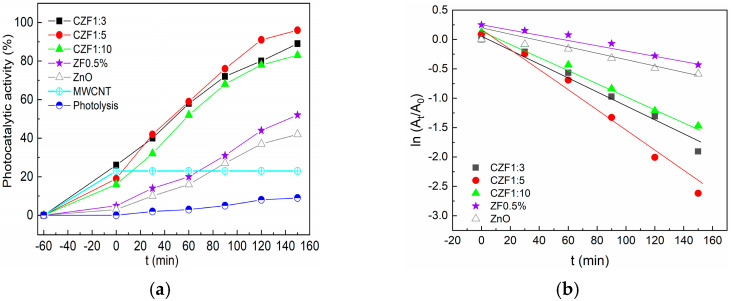
(**a**) Photocatalytic activity of the samples under UV irradiation against RhB. (**b**) Photodegradation kinetics.

The photocatalytic activity was tested on a synthetic solution of RhB under UV light irradiation. Before starting the photodegradation process, the solutions with a given concentration of photocatalysts were kept in the dark for 60 min until adsorption-desorption equilibrium was achieved. The adsorption increases with the increase in the MWCNT content in the composite samples. This may be due to the electrostatic interaction between the positively charged MWCNT and positively charged pollutant molecules [20]. After adsorption, the samples were irradiated with 365 nm UV light for 3 h. The photodegradation efficiency of the composite samples compared to the ZF0.5% is shown in Figure 8a. In addition, this figure contains the photocatalytic activity of ZnO, MWCNT and RhB photolysis. The best photocatalytic activity, 96%, was shown by the CZF1:5 sample.

The photodegradation kinetics of RhB were analyzed by applying the first kinetic order model. The equation describing this model is:

ln(A_t_ ⁄ A_0_) = −k_i_ × t (2)

where A_t_ and A_0_ represent the absorbance of RhB at time t and in the dark, respectively, k_i_ is the apparent rate constant. The plots obtained are shown in Figure 8b, with a linear dependence in t being observed. The apparent rate constant, k_i_, and the correlation coefficients R^2^ were obtained from the linear fitting of the dates and are presented in Table 4. These results support the conclusion that the CZF1:5 sample has the best photocatalytic activity.

Some works about organic pollutant degradation using MWCNT-based composite nanomaterials are presented in Table 5. Previously, similar results were obtained, but direct comparison is difficult due to the differences in the photocatalyst concentration, the concentration of initial pollutant, the UV lamp power and the irradiation time.

**Figure 9:**
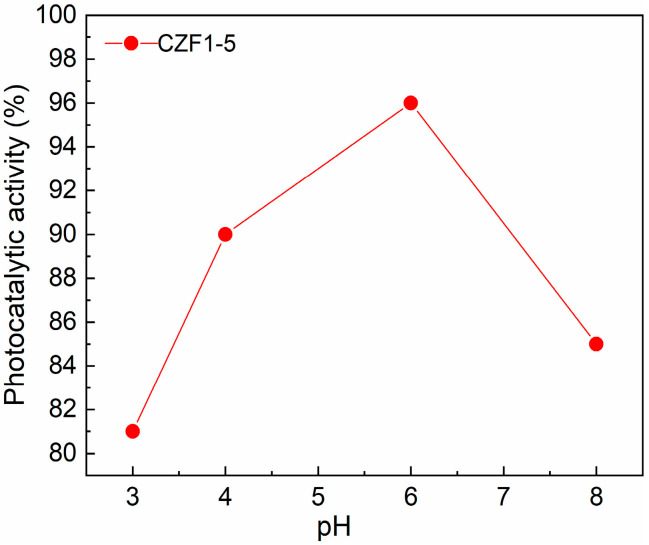
Effect of initial solution pH on pollutant photodegradation.

One of the parameters influencing the degradation efficiency of pollutants is the solution pH. Thus, the degradation efficiency of RhB using the CZF1:5 sample was tested by varying the pH between three and eight and maintaining a constant photocatalyst concentration (0.2 mg/mL). The results are presented in Figure 9. It can be observed that a pH value of six assures the best photocatalytic degradation. For higher pH, the degradation efficiency decreases, probably due to the repulsion between negatively charged pollutant molecules and nanoparticles [9]. In contrast, by utilizing an acidic solution (pH of about 3), the lowest photocatalytic activity was obtained, probably caused by the slight dissolution of the catalyst [54].

**Figure 10:**
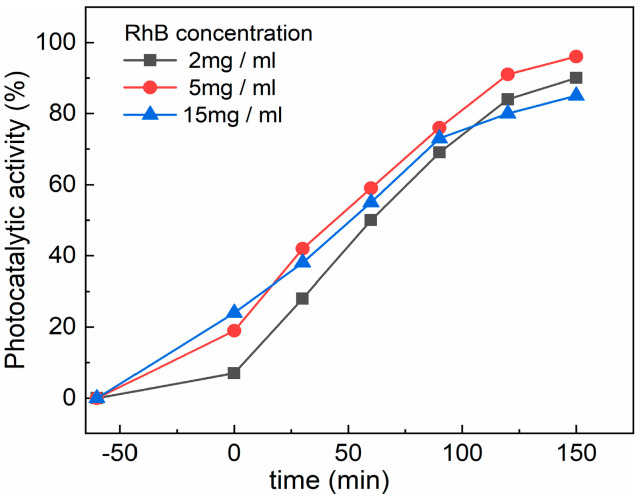
Effect of RhB concentration on the CZF1:5 sample photocatalytic activity.

The effect of the RhB concentration on the CZF1:5 sample’s photocatalytic activity was studied using three concentrations of RhB (2 mg/mL, 5 mg/mL and 15 mg/mL), and is shown in Figure 10. It can be observed that the adsorption capacity of the sample increases with the increase in the RhB concentration solution. This behavior can be explained based on the increased number of dye molecules from a diluted solution to a concentrated one. After 150 min irradiation, the 5 mg/ml solution was degraded the most, followed by the 2 mg/mL solution, and the lowest degradation was obtained for the 15 mg/L solution.

**Figure 11:**
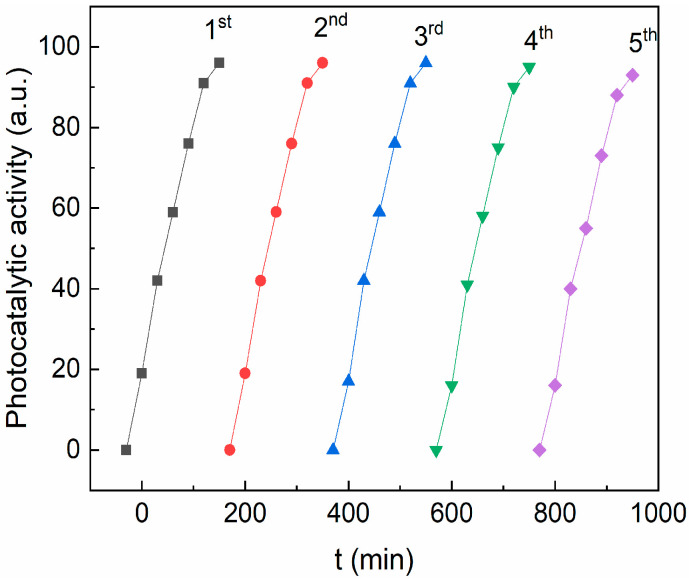
The reusability of CZF1:5 sample for degradation of RhB dye for five cycles under UV irradiation.

To evaluate the stability of the photocatalyst, several consecutive RhB photodegradation cycles were performed. Between two photodegradation cycles, the pollutant solution was removed, and the sample was washed with ethanol/water and dried overnight. Figure 11 shows the photocatalytic activity after five runs using the CZF1:5 sample as a photocatalyst. It can be observed that the sample efficiency remains practically unmodified after five photodegradation cycles and sustained the good stability of the sample. In addition, the CZF1-5 sample stability was verified through FT-IR (Figure S3, Table S1). No modification of the spectrum was observed after 150 min of UV irradiation.

**Figure 12:**
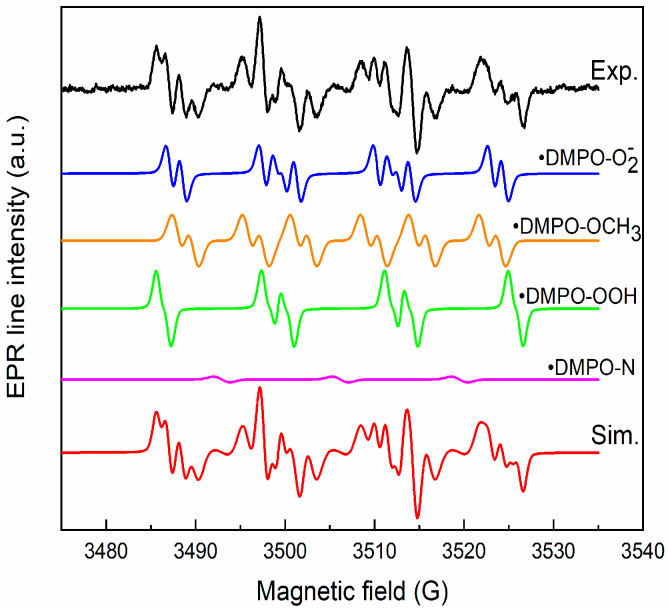
Experimental and simulated spectra of DMPO spin adducts generated by CZF1:5 sample after 25 min irradiation.

To elucidate the mechanism of RhB pollutant photodegradation, we investigated the generation of reactive oxygen species by the CZF1:5 sample under the action of UV light. We used the ESR, coupled with the spin trapping technique and DMPO (5,5-Dimethyl-1-pyrroline N-oxide), as a spin trapper. No ESR signal was obtained for the unirradiated sample, but a complex spectrum was obtained after 25 min of irradiation, as seen in Figure 12. The simulation was performed to identify each component of the spectrum.

The simulation results reveal that the spectrum is composed of the superposition of the following components: •DMPO-OCH_3_ (a_N_ = 13.2 G, a_H_ = 7.8 G, a_H_ = 1.6 G, relative concentration 38%), •DMPO-OOH (a_N_ = 13.8 G, a_H_ = 11.8, a_H_ = 0.9 G, g = 2.0098, relative concentration 29%), •DMPO-O_2_^−^ (a_N_ = 12.8 G, a_H_ = 10.4 G, a_H_ = 1.4 G, relative concentration 31%) and •DMPO-N (a_N_ = 13.9 G, relative concentration 2%). The •DMPO-OCH_3_ adduct spin is produced via the interaction of hydroxyl radicals (•OH) with DMSO solvent, and •DMPO-OOH is a result of •O_2_^−^ radicals protonation. The •DMPO-N appears through the cleavage of the N–C bond in DMPO [55]. These results indicate that the hydroxyl and superoxide radicals are generated under irradiation.

**Figure 13:**
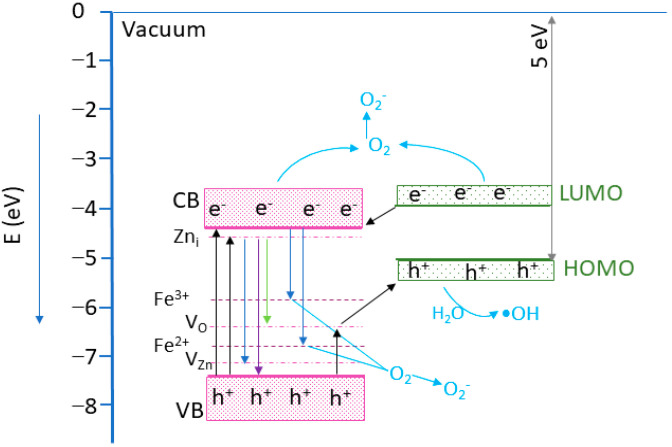
The proposed photocatalytic mechanism based on the energy band structure. The energy level positions were draw according to [33,56,57,58,62].

To explain the photocatalytic mechanism, a band alignment corresponding to the nanocomposite was proposed, considering the Eg values estimated by the UV-VIS and ZnO VB energy position of −7.44 eV [56]. For the MWCNT, an ionization energy of 5.01 eV was considered [57,58]. Analyzing the band edge energies, it can be observed that a type-II heterostructure was formed [56]. The proposed photocatalytic mechanism is illustrated in Figure 13. Following the UV light excitation of the composites, the electrons from the ZnO VB are promoted into the CB, generating holes in the VB. The photogenerated electrons from the LUMO band of the MWCNT can be transferred towards ZnO CB [56]. The electrons from ZnO CB can reach the photocatalyst surface, where interacting with adsorbed O_2_ will create •O_2_^−^ species. However, if the electrons have enough energy, they can attend the Fe^3+^ ions from the nanoparticle surface, generating Fe^2+^ ions. As Fe^2+^ ions are unstable, they will interact with the O_2_ molecules, forming Fe^3+^ and O_2_^−^ reactive species. In addition, Fe^2+^ ions can receive and electron form unstable Fe^1+^ ions, which will further interact with the O_2_ molecules, generating O_2_^−^ and Fe^2+^ ions [33]. In addition, the photogenerated electrons can move to the Zn_i_ levels, followed by transitions towards the V_O_ and V_Zn_ levels, leading to radiative emissions [42,59]. The remaining holes in the ZnO VB will pass to the MWCNT via impurity levels in the ZnO bandgap and can interact with the H_2_O molecules, generating •OOH reactive species [33]. Consequently, the MWCNT’s role is to efficiently separate the photogenerated charges, allowing electrons to generate ROS species and increasing the photocatalytic efficiency [60]. The photocatalytic investigation reveals the existence of an optimum amount of ZF0.5%, which assures the best photocatalytic efficiency. A higher concentration of ZF0.5% nanoparticles on the MWCNT surface could prevent incident light from reaching the photocatalyst’s active surface, leading to a decrease in the photocatalyst efficiency [61].

**Figure 14:**
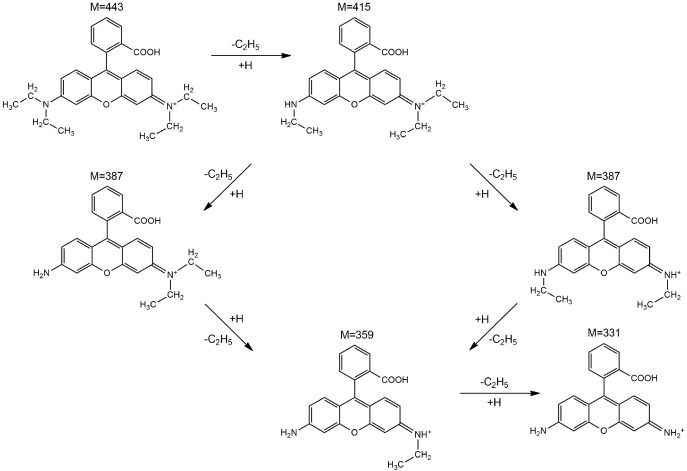
The proposed photodegradation pathways of RhB based on the intermediates identified by the HPLC–MS method.

To better understand the photodegradation mechanism, the intermediates produced by irradiating the RhB solution for 150 min in the presence of the sample with the best photocatalytic activity (CZF1-5) were identified through HPLC/MS (Figures S4 and S5). The fragmentation of RhB with a *m*/*z* value of 443 generates five major N-de-ethylated intermediates, including N, N-diethyl-N′-ethylrhodamine (*m*/*z* = 415), N, N-diethylrhodamine/N′-ethyl-N-ethylrhodamine (*m*/*z* = 387), N-ethylrhodamine (*m*/*z* = 359) and rhodamine (*m*/*z* = 331). Consequently, the photodegradation of RhB occurs through the N-de-ethylation process, which is schematically shown in Figure 14.

The sample with the best photocatalytic efficiency (CZF1-5) was tested on an antibiotic (oxytetracycline) degradation to demonstrate the sample’s ability to degrade other types of pollutants (Figure S5). The obtained degradation efficiencies were 85% (by monitoring the 360nm absorption peak) and 30% (by monitoring the 270 nm absorption peak). A longer irradiation time would likely have been necessary to improve the photocatalytic performance.

## 4. Conclusions

In this work, MWCNTs decorated with 0.5%Fe-doped ZnO nanoparticles were prepared through polymer wrapping using poly(allylamine hydrochloride). Three samples, in which the molar ratio between the MWCNT and ZF0.5% was 1:3, 1:5 and 1:10, were prepared. XRD diffraction certified the formation of composite samples, evidencing the patterns specific to ZnO, with a hexagonal structure, and that specific to MWCNTs. From the EPR spectra analysis results, ZF0.5% presents three types of paramagnetic species: isolated Fe^3+^ ions, ferromagnetically coupled Fe^3+^ ions and oxygen vacancies. The TEM images show a tubular shape of the MWCNT decorated with ZF0.5% nanoparticles with a star-like morphology. The band gap energies of the samples decrease with the increase in the MWCNT content. The samples show emissions in both the UV and visible ranges. The emissions from UV are known as the near band edge, and that from the visible range are associated with the emission caused by different defect states, such as Zn in interstitial sites or oxygen vacancy. Both types of emissions are quenched with the increase in the MWCNT content due to a delay in the recombination process induced by the charge transfer between the defect levels of the ZnO nanoparticles and MWCNT. All of the samples show photocatalytic activity against RhB under UV irradiation. The best photocatalytic activity was obtained for the CZF1:5 sample, and this sample’s stability was proven by performing five consecutive cycles. Through HPLC characterization, it was evidenced that the photodegradation of RhB occurs through the N-de-ethylation process. Analyzing the ROS generated by the CZF1:5 sample under UV irradiation shows that hydroxyl and superoxide radicals are involved in the photocatalytic reactions. The photocatalytic mechanism was elucidated based on the ROS generated under UV irradiation and in correlation with the structural and optical properties.

## Figures and Tables

**Table 1:** The lattice parameters, unit cell volume, mean crystallites size corresponding to ZnO crystalline phase and the quality parameters for the Rietveld refinement.

Sample	a = b (Å)	c (Å)	V (Å^3^)	D_cryst_ (nm)	R_wp_ (%)	R_p_(%)	R_exp_(%)	χ^2^
ZnO	3.2472	5.2023	47.508	13.4	4.74	3.55	3.15	1.5
ZF0.5%	3.2480	5.2060	47.565	11.5	7.31	5.42	5.14	1.6
CZF1:3	3.2478	5.2059	47.560	11.5	10.84	8.39	7.41	1.7
CZF1:5	3.2479	5.2059	47.562	11.5	9.83	7.38	6.42	1.6
CZF1:10	3.2479	5.2060	47.563	11.5	8.21	6.83	5.98	1.6

**Table 2:** The area (A_peak_) of the deconvoluted PL peaks of Figure 5.

Sample	A_3.86_	A_3.50_	A_3.30_
CZF1:10	0.73	3.15	4.37
CZF1:5	0.46	1.13	2.55
CZF1:3	0.82	2.28	2.88

**Table 3:** The area (A_peak_) of the deconvoluted PL peaks of Figure 6.

Sample	A_3.02_	A_2.82_	A_2.62_	A_2.50_	A_2.27_
CZF1:10	3.63	-		1.86	8.39
CZF1:5	2.56	1.94	1.18	1.58	6.56
CZF1:3	0.40	0.20		1.32	1.66

**Table 4:** The apparent rate constant, k_i_, and the correlation coefficients R^2^.

Sample	k_i_ × 10^−3^ (min^−1^)	R^2^
ZnO	4.1	0.98
ZF0.5%	4.6	0.98
CZF1:3	12.6	0.96
CZF1:5	20.16	0.99
CZF1:10	11.2	0.96

**Table 5:** Some works related to pollutant photodegradation using MWCNT-based photocatalysts.

Photocatalyst	Pollutant Type	Pollutant Concentr.	Light Irrad./Power Source or Illuminance	Irrad. Time (min)	Catalyst Concentr(mg/mL)	Removal Rate (%)	Ref
MWCNT-ZnO:Fe	RhB	1 × 10^−5^ M	UV/30 W	150	0.2	95	This work
g-C_3_N_4_/ZnO/MWCNT	Alprazolam	0.1 mmol/L	UV/125 W	15	1.0	100	[50]
ZnO-Ag/MWCNT	Phenol	10 mg/l	UV/not specified	240	1.0	81	[51]
NiFe_2_O_4_/MWCNT	Methylene blue	20 mg/l	UV/233 lux	150	0.025	88	[52]
Bi_0.5_Na_0.5_TiO_3_/MWCNT	RhB	5 mg/L	UV/300 W	40	1.0	100	[53]

## Data Availability

The data presented in this study are available on reasonable request.

## Supplementary Materials

The following supporting information can be downloaded at: https://www.mdpi.com/article/10.3390/ma16072858/s1.
