# Supplementary material for: Photoluminescence and Photocatalytic Properties of MWNTs Decorated with Fe-Doped ZnO Nanoparticles
Source: Materials (Basel). 2023 Apr 3;16(7):2858. doi: 10.3390/ma16072858 (PMC10095740; doi:10.3390/ma16072858)
Supplement: Supplementary file 1 [file materials-16-02858-s001.zip › materials-2303319-supplementary.pdf]

Photoluminescence and photocatalytic properties of MWCNT decorated with Fe doped ZnO nanoparticles

Adriana Popa [1], Maria Stefan [1], Sergiu Macavei [1], Laura Elena Muresan [2], Cristian Leostean [1], Cornelia Veronica Floare-Avram [1], Dana Toloman [1,*]

[1] National Institute for Research and Development of Isotopic and Molecular Technologies, 67-103 Donat, 400293, Cluj-Napoca, Romania; adriana.popa@itim-cj.ro (A.P.); cristian.leostean@itim-cj.ro(C.L.)
[2] "Raluca Ripan" Institute for Research in Chemistry, "Babes-Bolyai" University, 30 Fântânele, 400294 Cluj-Napoca, Romania; laura.muresan@ubbcluj.ro
* Correspondence: dana.toloman@itim-cj.ro

1. Influence of Fe doping degree on the photocatalytic performance of ZnO nanoparticles

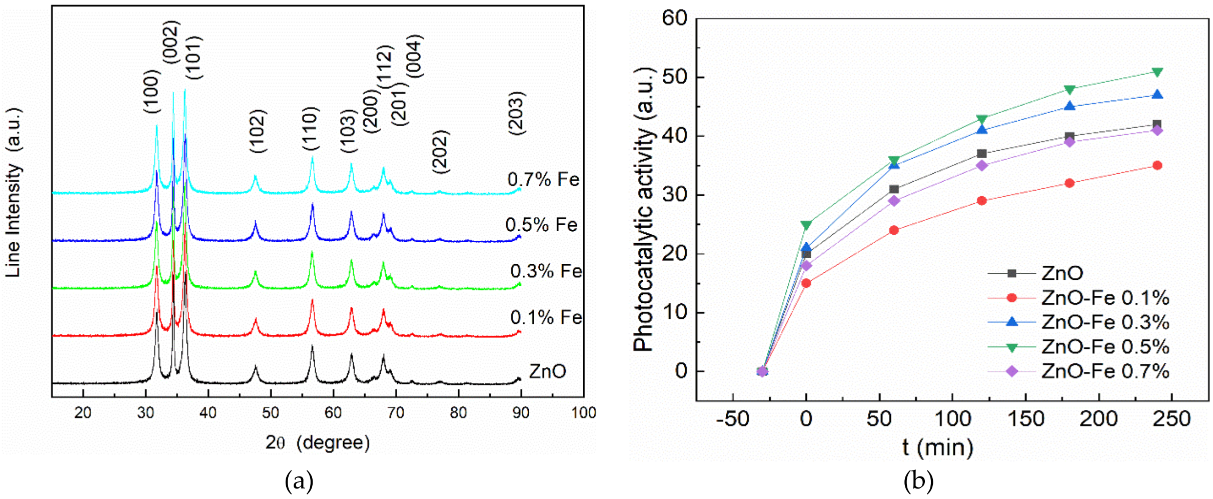

Figure S1 (a) XRD of $Zn_{1-x}Fe_xO$ nanoparticles and (b) the photocatalytic activity of the samples

2. Size distribution of ZF0.5% nanoparticles evaluated by TEM

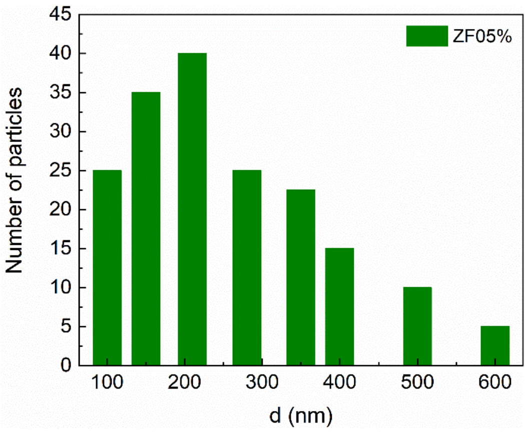

Figure S2. Size distribution of ZF05% nanoparticles estimated from TEM images

3. FT-IR spectra of CZF1-5 before and after photodegradation

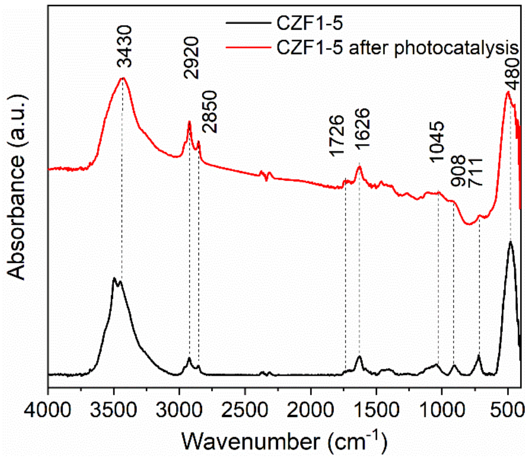

Figure S3. FT-IR spectra of CFZ1-5 before and after photodegradation

Table S1. FT-IR band assignment

FT-IR bands (cm^{-1})	Band assignment
3430	O-H
2920/ 2850	C–H stretching vibrations
1726	C = O
1626	C = C
1045	C–O stretching vibration
908	Zn–O–C bending vibration
711	hexagonal carbon in pristine MWCNTs
480	Zn-O

4. HPLC-MS Characterization

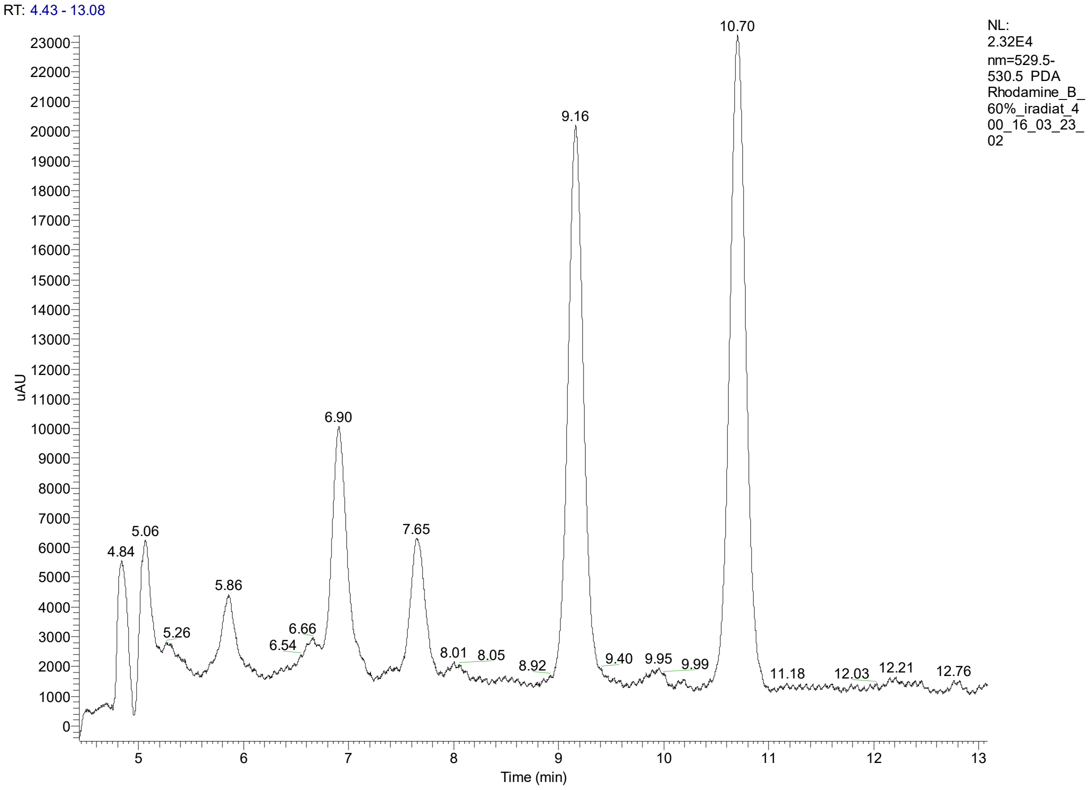

Figure S4. HPLC-MS Chromatogram of RhB solution in the presence of CZF1-5 sample after 150min UV light irradiation

Mass spectrum for the retention time 5.06 min

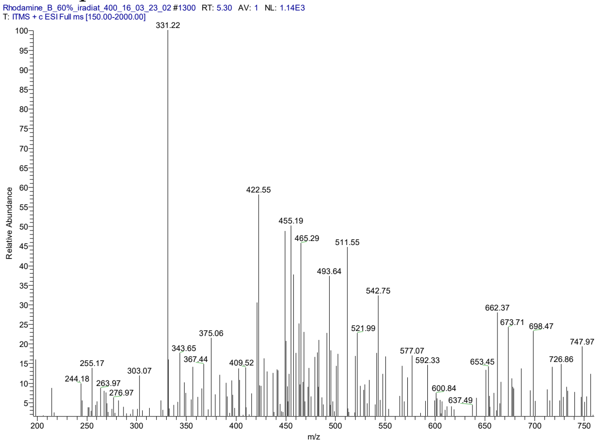

Mass spectrum for the retention time 5.86 min

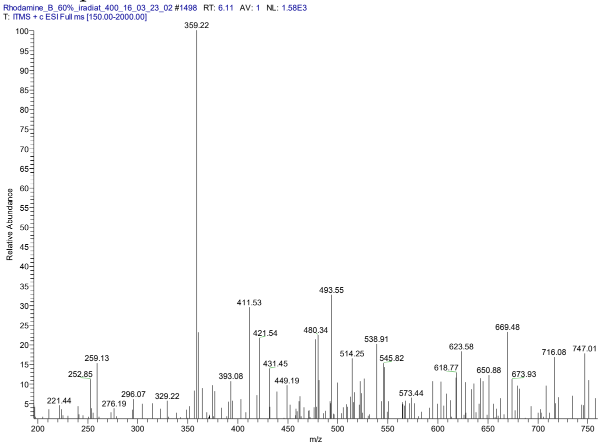

Mass spectrum for the retention time 6.90 min

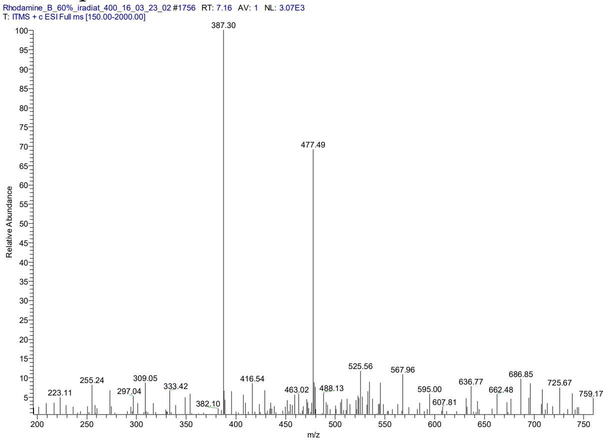

Mass spectrum for the retention time 7.65 min

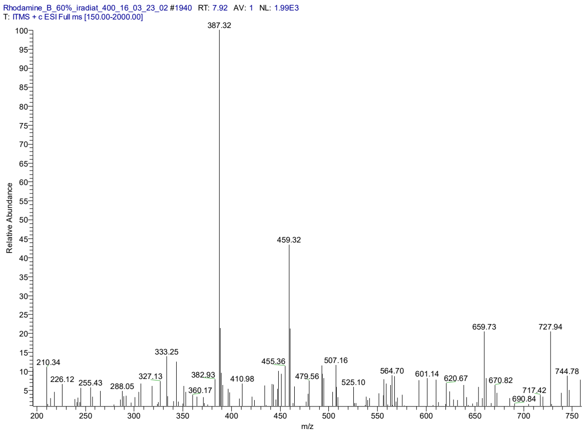

Mass spectrum for the retention time 9.16 min

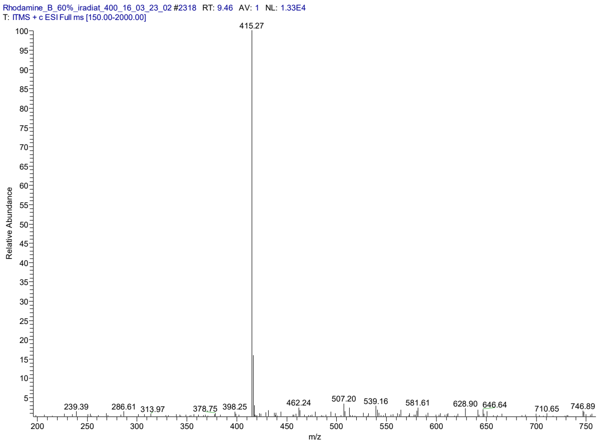

Mass spectrum for the retention time 10.70 min

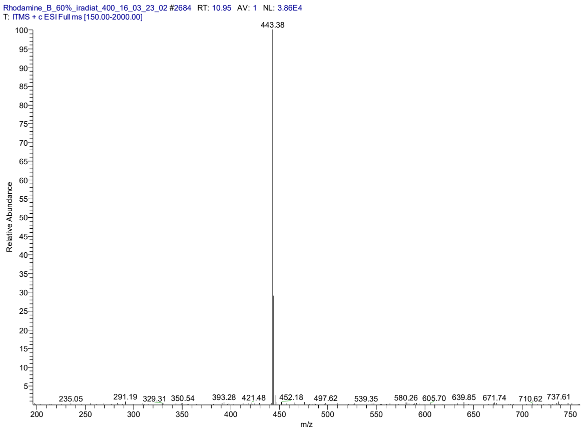

Figure S5. Mass spectrum for different retention times

5. Oxytetracycline photodegradation

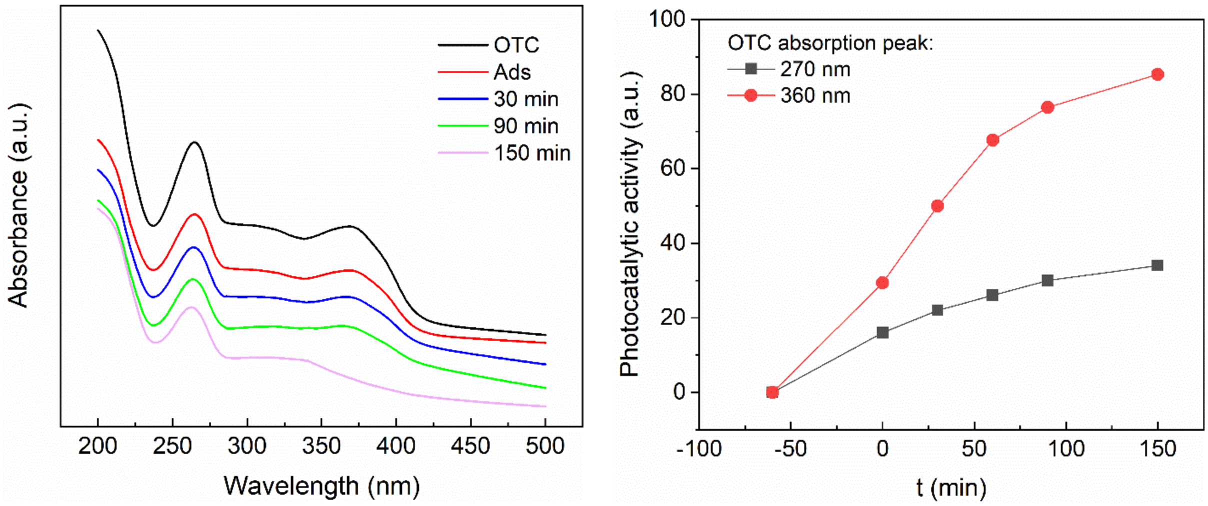

Figure S6 Photocatalytic degradation of Oxytetracycline in the presence of CZF1-5 nanocomposites
